# Recurrent myocarditis-like episodes in a patient with a rare variant in *DES* gene: an uncommon hot-phases cardiomyopathy

**DOI:** 10.1093/eschf/xvag030

**Published:** 2026-01-19

**Authors:** Laura Manfrin, Antonio Parlati, Valeria Novelli, Giulia Corona, Alessia Paldino, Marco Castronuovo, Georgette Khoury, Nicola Gonano, Andrea Baggiano, Luca Martini, Fiorella Puttini, Gianfranco Sinagra, Piergiuseppe Agostoni, Massimo Mapelli

**Affiliations:** Centre of Diagnosis and Management of Cardiomyopathies, Azienda Sanitaria Universitaria Giuliano Isontina, University of Trieste, Italy; Heart Failure Unit, Centro Cardiologico Monzino, IRCCS, Via Parea 4, 20138 Milan, Italy; Heart Failure Unit, Centro Cardiologico Monzino, IRCCS, Via Parea 4, 20138 Milan, Italy; Unità di Genetica Cardiovascolare, Centro Cardiologico Monzino, IRCCS, Milan, Italy; Unità di Genetica Cardiovascolare, Centro Cardiologico Monzino, IRCCS, Milan, Italy; Centre of Diagnosis and Management of Cardiomyopathies, Azienda Sanitaria Universitaria Giuliano Isontina, University of Trieste, Italy; Azienda Ospedaliera ‘S. Maria’, Terni, Italy; Azienda Ospedaliera ‘S. Maria’, Terni, Italy; Centre of Diagnosis and Management of Cardiomyopathies, Azienda Sanitaria Universitaria Giuliano Isontina, University of Trieste, Italy; Heart Failure Unit, Centro Cardiologico Monzino, IRCCS, Via Parea 4, 20138 Milan, Italy; Department of Clinical Sciences and Community Health, Cardiovascular Section, University of Milan, Via Parea 4, Milan 20138, Italy; Heart Failure Unit, Centro Cardiologico Monzino, IRCCS, Via Parea 4, 20138 Milan, Italy; Heart Failure Unit, Centro Cardiologico Monzino, IRCCS, Via Parea 4, 20138 Milan, Italy; Centre of Diagnosis and Management of Cardiomyopathies, Azienda Sanitaria Universitaria Giuliano Isontina, University of Trieste, Italy; Heart Failure Unit, Centro Cardiologico Monzino, IRCCS, Via Parea 4, 20138 Milan, Italy; Azienda Ospedaliera ‘S. Maria’, Terni, Italy; Heart Failure Unit, Centro Cardiologico Monzino, IRCCS, Via Parea 4, 20138 Milan, Italy; Department of Clinical Sciences and Community Health, Cardiovascular Section, University of Milan, Via Parea 4, Milan 20138, Italy

**Keywords:** Desmin (DES), Desminopathy, Non-dilated left ventricular cardiomyopathy (NDLVC), Myocarditis, Hot-phases cardiomyopathy, Arrhythmogenic cardiomyopathy (ACM)

## Introduction

Genetic alterations in *DES* gene, encoding for desmin—a muscle-specific intermediate filament (IF) protein—are associated with a broad phenotypic spectrum including cardiomyopathies, conduction abnormalities, and skeletal myopathy.^1^ An association between *DES* variants and myocardial inflammation has not been clearly established. Acute myocarditis and hot-phase cardiomyopathies can mimic acute coronary syndromes (ACSs),^2,3^ and in such scenarios, cardiac magnetic resonance (CMR) plays a crucial role in differential diagnosis, particularly in distinguishing ischaemic from non-ischaemic causes, such as Myocardial Infarction with Non-Obstructive Coronary Arteries (MINOCA) and Takotsubo syndrome. Genetic testing is not mandatory in acute myocarditis but it may be warranted in selected cases.^3^

## Case report

A 54-year-old Caucasian woman was referred to our centre after multiple episodes of MINOCA. Her medical history included subclinical hyperthyroidism, colonic diverticulosis, and a prior hysterectomy. She was a former smoker with no other cardiovascular risk factors. Her mother had a history of dilated cardiomyopathy (DCM), but there was no family history of sudden cardiac death (SCD) or malignant arrhythmias.

**Figure 1:**
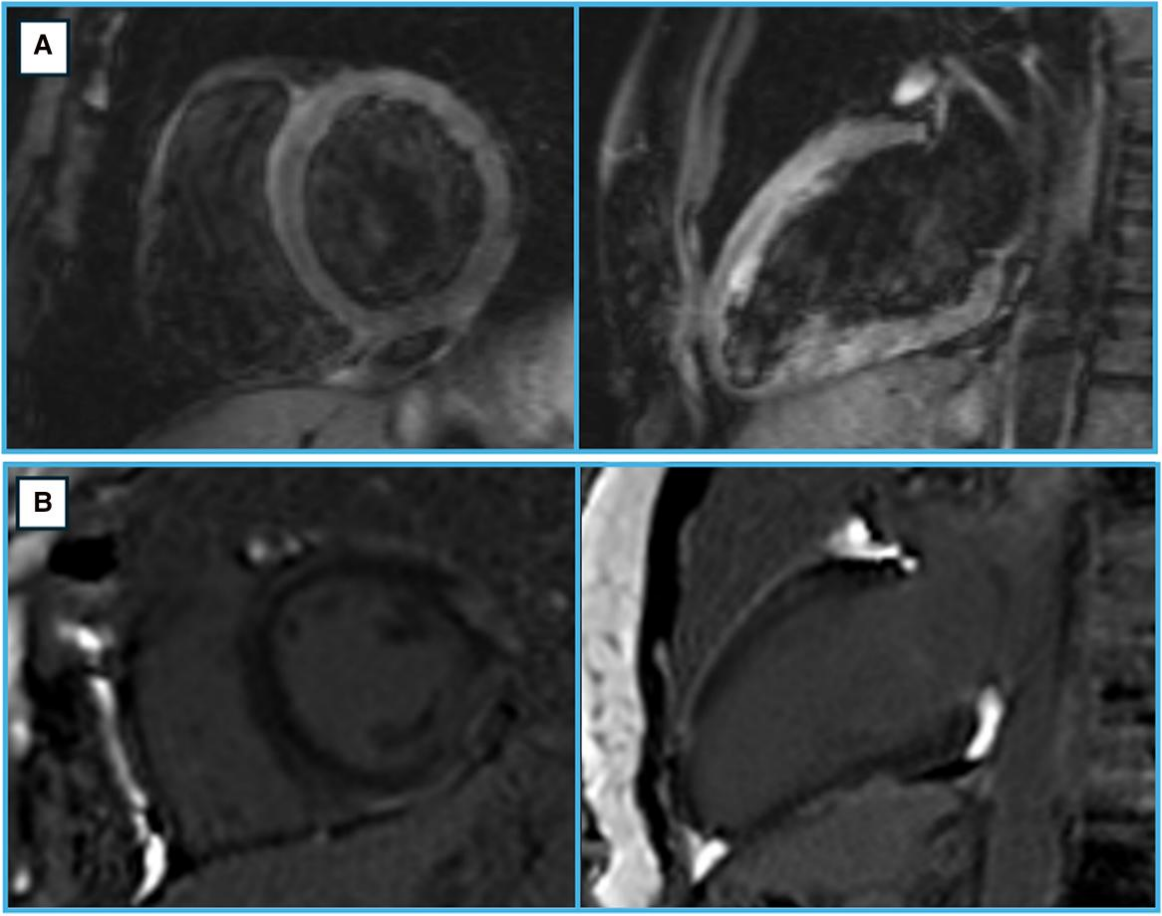
Cardiac magnetic resonance imaging during the acute phase of the first episode (2012). Basal short-axis and vertical long-axis T_2_-weighted sequences show signal hyperintensity in the basal and medial segments of the anterior and anterolateral walls (*A*). In the same segments LGE with a subepicardial and intramyocardial distribution is present, but it can be found also in the proximal and medial segments of the inferolateral and inferior walls. (*B*). These findings are consistent with myocardial oedema. 2018 Lake Louise Criteria for myocarditis are met. CMR, cardiac magnetic resonance; LGE, late gadolinium enhancement

The patient initially presented to a spoke centre in 2012 with acute-onset chest pain. Electrocardiogram (ECG) showed minimal repolarization abnormalities in leads DI and aVL. Troponin levels were elevated. Echocardiography revealed hypokinesis of the mid-basal anterior wall with preserved left ventricular ejection fraction (LVEF). Coronary angiography (CA) demonstrated normal coronary arteries, no invasive functional tests were performed. Cardiac magnetic resonance findings were consistent with myocardial inflammation in the basal and medial segments of the anterior and lateral walls (*Figure 1*). At the time, a presumptive diagnosis of MINOCA secondary to coronary vasospasm was made, and the patient was started on antiplatelet therapy, ranolazine, and a low dose of a statin and ACE inhibitor. She remained asymptomatic for the next 10 years, during this period she did not undergo follow-up CMR or cardiac enzyme monitoring.

**Figure 2:**
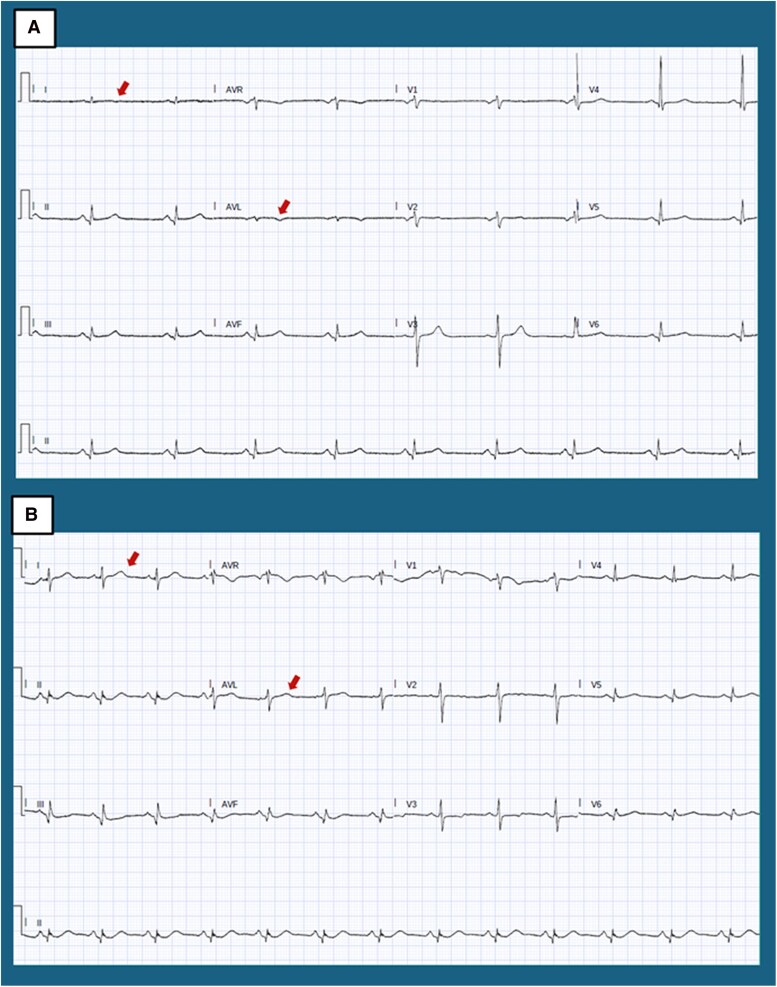
Comparison between electrocardiogram during the acute phase (*A*) and the stable phase (*B*). In both cases a sinus rhythm is present, with normal AV and IV conduction. Minimal q waves in the inferior leads can be appreciated. QRS voltages in the limb leads are not well represented, criteria for low voltages are not met though. During the acute phase transient repolarization abnormalities in DI and aVL can be observed, with a normalization during the stable phase

**Figure 3:**
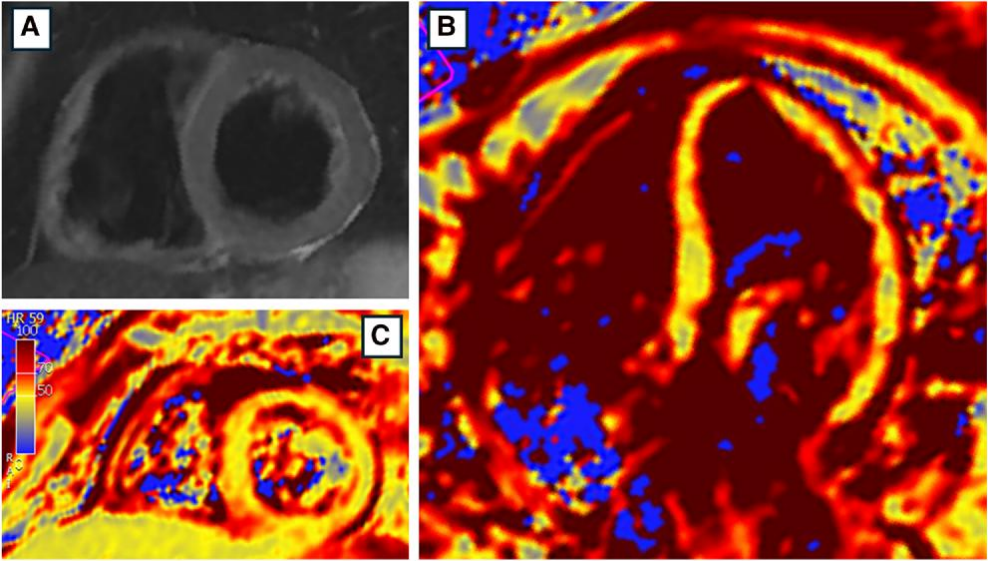
Cardiac magnetic resonance in the stable phase, after two myocarditis-like episodes (2022), oedema dedicated sequences. T2 weighted sequences (*A*) and T2 mapping (*B*, *C*) demonstrated no sign of oedema. T2 measured at basal septum (LGE absent): 49 ± 2 ms; T2 measured at anterolateral basal segment (LGE present): 47 ± 1 ms; Global T2: 48 ms (T2 normal reference: <55 ms). CMR, cardiac magnetic resonance

**Figure 4:**
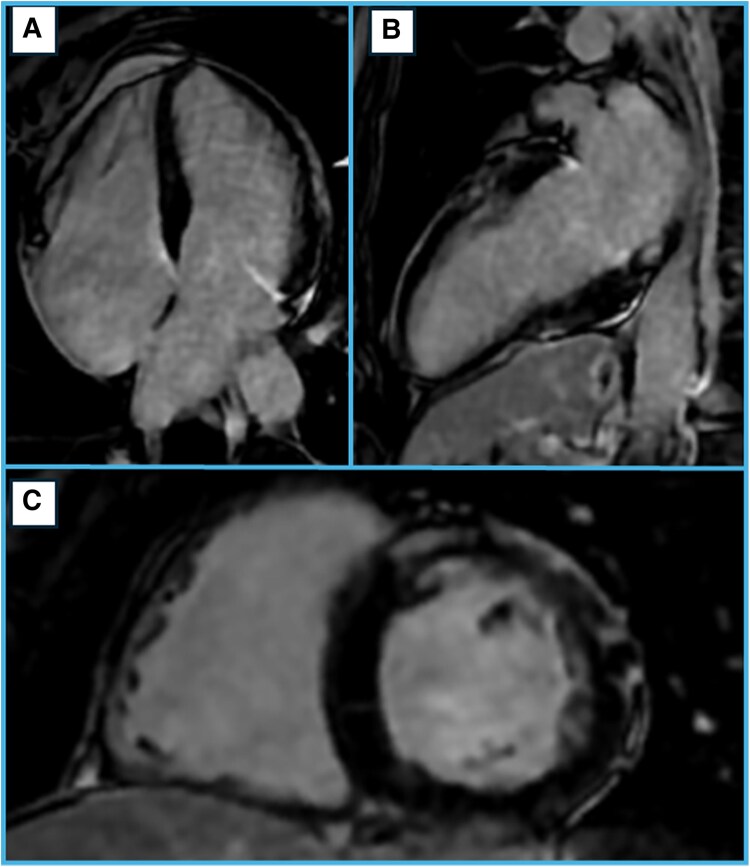
Cardiac magnetic resonance in the stable phase, after two myocarditis-like episodes (2022), LGE sequences. Medial short axis (*A*), horizontal long axis (*B*), and vertical long axis (*C*) images show extensive fibrosis with a non-ischaemic pattern: LGE is present with a subepicardial and intramyocardial distribution in the proximal and medial segments of the anterior, lateral and inferior walls. CMR, cardiac magnetic resonance; LGE, late gadolinium enhancement

In April 2022, the patient re-presented with exertional chest pain. Electrocardiogram showed T-wave inversions in lateral leads (*Figure 2*). High-sensitivity troponin I (hs-TnI) measurements followed a curve peaking at 17 388 ng/l [normal values (n.v.) <11 ng/l], BNP was 589 pg/ml. Echocardiography showed anterior hypokinesis with an LVEF of 45%. Repeat CA again showed no significant coronary disease. COVID-19 testing was negative. Cardiac magnetic resonance, performed 1 month later, showed normal volumes and ejection fraction of both the right and left ventricles. Dedicated sequences demonstrated the absence of oedema (*Figure 3*), but subepicardial and intramyocardial LGE were found in the proximal and medial segments of the anterior and lateral walls, as well as circumferential pericardial effusion (*Figure 4*).

**Figure 5:**
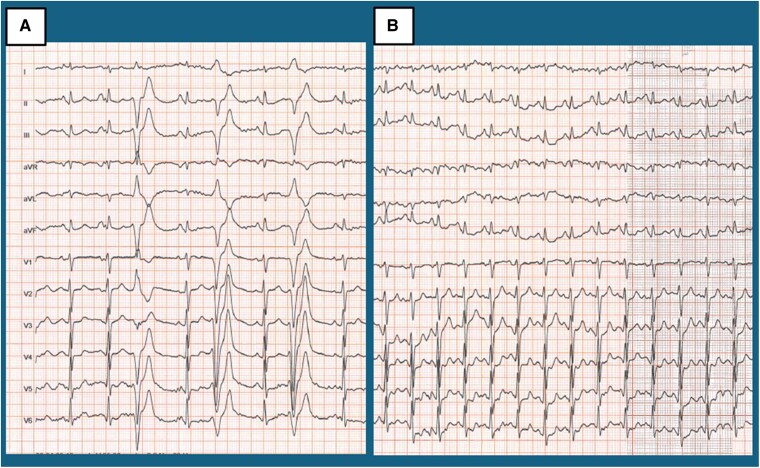
Electrocardiogram during stress test. During the first phase of exercise (*A*) an increase in the ventricular extrasystolic burden is observed, with polymorphic PVCs (RBBB and LBBB morphology both with superior axis). At the peak of the exercise (*B*) PVCs are suppressed. PVCs, premature ventricular contractions; RBBB, right bundle branch block; LBBB, right bundle branch block

In October 2022, she underwent routine follow-up, remaining asymptomatic with normal physical examinations and labs. A 24-h Holter monitor recorded a low burden of ectopic ventricular beats (EVBs), though an increased number of EVBs was observed during exercise testing (*Figure 5*). The cardiopulmonary exercise test demonstrated normal functional capacity, with early signs of cardiogenic limitation and/or deconditioning (peak oxygen intake 22.4 ml/kg/min, corresponding to 88% of the predicted). There were no signs of pulmonary vascular nor ventilatory limitations, and no significant arrhythmias were observed during the test. This second episode was interpreted as an acute myocarditis.

In March 2025, she was hospitalized again for chest pain after intense emotional stress. Electrocardiogram showed minimal repolarization abnormalities in the high lateral leads. Hs-TnI curve peaked at 4992 ng/l (n.v. <11 ng/l). Echocardiography revealed anterolateral hypokinesis with a reduced LVEF of 30%, which normalized prior to discharge. Coronary angiography was again normal, and inflammatory markers were persistently negative. Although the echocardiographic picture was not perfectly typical, the clinical picture was interpreted as Takotsubo syndrome.

**Figure 6:**
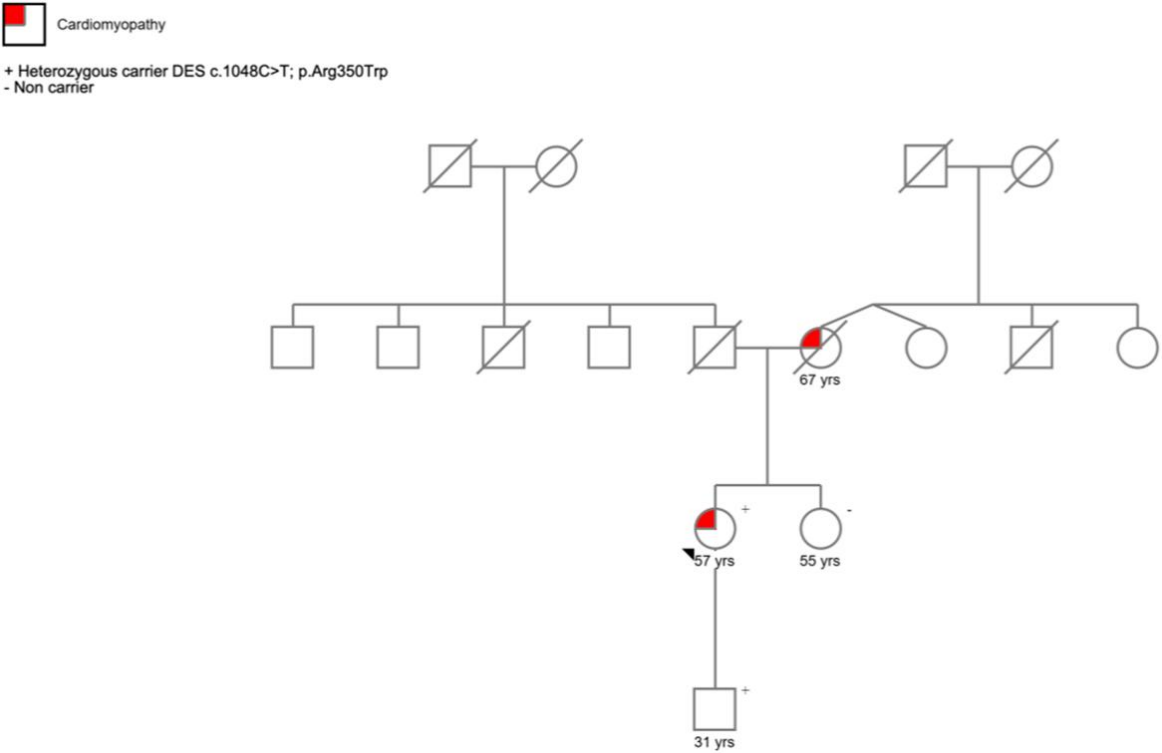
Pedigree of patient’s family. The proband is indicated with an arrow. Patient’s mother was affected by and died from DCM, although she did not undergo genetic testing. Proband’s variant was searched in her first-degree relatives: her sister, who does not carry the same variant, and her son, which tested positive. DCM, dilated cardiomyopathy

Considering the myocardial injury episodes recurrency and the family history positive for cardiomyopathy, a genetic testing by target sequencing (using a cardiomyopathy panel of 38 genes) was performed. Results showed a heterozygous missense variant in *DES* gene (c.1048C>T; p.Arg350Trp), classified as likely pathogenic (LP) according to the American College of Medical Genetics/Association of Molecular Pathology. The patient never experienced neuro-muscular symptoms and CK levels were always normal beside the acute phases. First-degree relatives, sister and son, had normal findings on ECG and echocardiography, but the same variant was detected in patient’s son (*Figure 6*).

Since the myocarditis-like episodes resolved spontaneously and rapidly, and the patient remained completely asymptomatic, free of arrhythmias, and showed no signs of latent myocardial inflammation (such as oedema on CMR or persistent troponin elevation) during periods of clinical stability, an endomyocardial biopsy was not performed and immunosuppressive therapy was not initiated, either during the acute episodes or the stable phases. After the 2022 episode, antiplatelet therapy and ranolazine were interrupted, while ACE inhibitor was continued. A low dose of beta-blocker and a mineralocorticoid receptor antagonist were started.

By now, the patient has not been implanted with an internal or subcutaneous cardiac defibrillator, since despite a close monitoring with serial 24 h Holter ECGs and stress tests, significant arrhythmias were never detected. Furthermore, LVEF during the stable phases was never below 35% and other clinical ‘red flags’ were absent, such as unexplained syncope or a positive familiar history for SCD. Finally, *DES* is not included in the list of genotypes associated with high risk of SCD.^4^

## Discussion

This clinical case highlights how, despite multiple investigations, differential diagnosis of pathologies that fall under an initial MINOCA definition can be complex. Genetics contribution, albeit with an unconventional mutation, was found to be fundamental. The three acute episodes were retrospectively re-evaluated, and a common aetiology has been investigated. The patient consistently presented with findings suggestive of ACS, but CA and CMR definitively excluded an ischaemic aetiology. During her first event in 2012, she underwent CMR during hospitalization, which revealed transmural myocardial oedema and non-ischaemic LGE. A retrospective revision of clinical presentation, laboratoristic and CMR findings supported an acute myocarditis diagnosis rather than coronary vasospasm, and it seems reasonable to consider the following as possible relapses. The 2022 and 2025 episodes followed a similar clinical pattern. In 2022, CMR imaging was performed one month after the acute event and showed extensive subepicardial LGE in a non-coronary distribution and pericardial effusion. The delay between the acute episode of myocardial damage and the execution of CMR imaging can explain the absence of myocardial oedema, being well established that CMR has higher sensitivity in detecting active myocardial inflammation when performed within the first 2 weeks of symptom onset.^5^ The detection of LGE after the resolution of the acute phase also excluded a diagnosis of stress cardiomyopathy.^5^ According to the phenotypic classification of cardiomyopathies, the presence of non-ischaemic myocardial fibrosis on CMR with a non-dilated left ventricle (LV) supports a non-dilated left ventricular cardiomyopathy (NDLVC) diagnosis.^4^

Genetic testing, which yields both diagnostic and prognostic implications for probands and family members, revealed a heterozygous missense variant in the *DES* gene (p.Arg350Trp), reinforcing a cardiomyopathy diagnosis. Desmin is a muscle-specific IF protein, which interacts with multiple cell elements, and plays a critical role in maintaining mechanical integrity and cellular architecture. Autosomal dominant is the most frequent inheritance pattern, and cardiac phenotype can manifest with all types of cardiomyopathies.^1^ Recent studies recognized *DES* variants as a rare cause of NDLVC, and adverse clinical outcomes, including SCD and malignant Vas, were reported.^6^ p.Arg350Trp is a rare variant which lies within the central rod domain, a highly conserved region where most of P/LP *DES* mutations have been identified.^1,7^ R350P mutant desmin, exerting dominant-negative effect, alters the assembly of IF resulting in cytoplasmic aggregates in affected tissues.^7^

Acute myocarditis has traditionally been considered an acquired condition, usually triggered by viral infections. Growing evidence suggests a genetic predisposition especially in patients with recurrent episodes, a family history of cardiomyopathy, or suggestive findings on CMR.^2^

Hot-phase cardiomyopathy and genetic-positive myocarditis have been predominantly associated with desmosomal gene mutations, such *DSP*.^8^ Pathogenic (P)/LP variants in this context have been related to a higher risk of adverse outcomes^8^ but a possible favourable role of immunosuppressive therapy on prognosis is emerging.^9^ Myocarditis-like presentation is not the usual form of cardiac involvement in desminopathies, though an accurate literature revision revealed interesting evidence supporting this association.^6,10^

**Figure 7:**
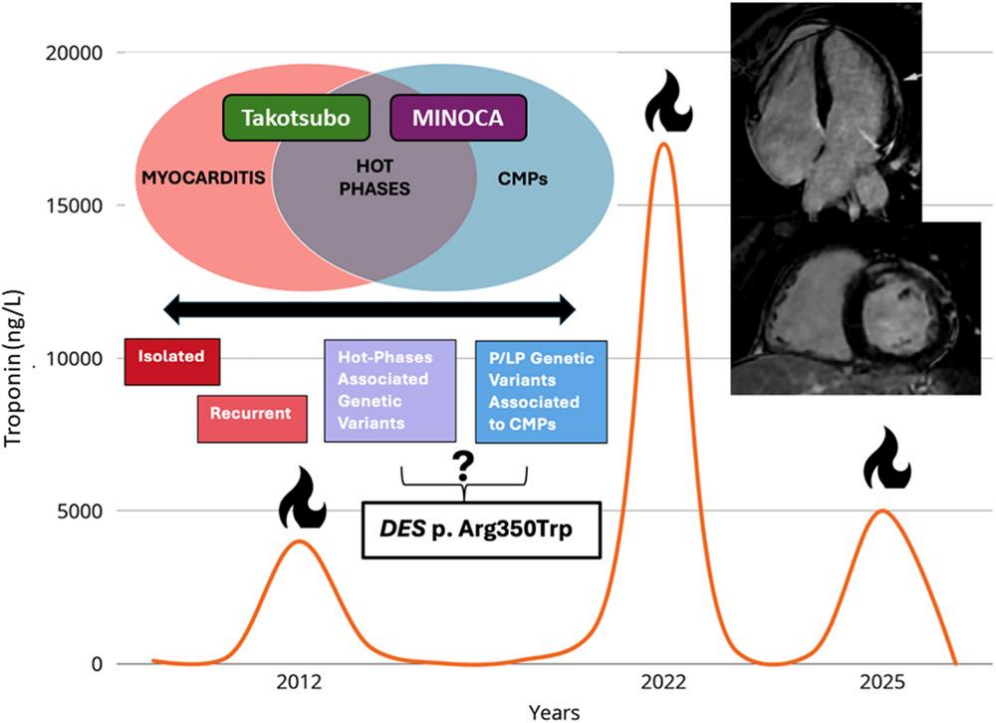
Timeline of the patient's clinical history over several years. The figure illustrates the longitudinal course of the disease, highlighting the recurrence of acute inflammatory ‘hot phases’ (marked by fire symbols), each associated with a rise in high-sensitivity troponin levels (ng/l). These episodes occurred in the absence of significant coronary artery disease and were variably labelled as MINOCA, myocarditis, or Takotsubo syndrome based on the clinical and imaging findings. The diagnostic re-evaluation over time is shown in parallel with symptom recurrence and biomarker trends. MINOCA, myocardial infarction with non-obstructive coronary arteries; CMPs, cardiomyopathies; P/LP, pathogenetic/likely pathogenetic

Genetic, molecular and clinical evidence is coherent with the phenotypic expression of myocardial damage resulting in fibrosis, and a possible concomitant role of inflammation in *DES*-associated cardiomyopathy is emerging (*Figure 7*). This case underscores how, despite many efforts to investigate the pathophysiological and clinical mechanisms underlying cardiomyopathies, it remains challenging to accurately label complex clinical pictures. The rarity of these syndromes contributes to the difficulty in structuring randomized clinical trials to evaluate therapy outcomes.

### Conclusion

The overlap between myocarditis and cardiomyopathies poses a significant diagnostic challenge. We report the case of a rare *DES* variant carrier who experienced recurrent episodes of acute myocardial injury exiting in extensive myocardial fibrosis. Our findings suggest that myocardial inflammation could be part of the wide spectrum of desminopathies clinical presentations and support broader adoption of genetic testing in patients with recurrent myocarditis-like presentations and family history of cardiomyopathy. Further research is needed to better understand the pathophysiology of *DES* variants and to improve the management of affected patients and healthy carriers.

## Data Availability

No data were generated or analysed for this manuscript.
